# Association between riboflavin intake and the risk of all-cause mortality of patients with chronic kidney disease: A retrospective cohort study

**DOI:** 10.1097/MD.0000000000039417

**Published:** 2024-09-27

**Authors:** Xiaoxu Ren, Rong Wang, Fen Liu, Quanzhen Wang, Hairong Chen, Yunfeng Hou, Lifeng Yu, Xiangchun Liu, Zhiming Jiang

**Affiliations:** a Department of Nephrology, Shandong Provincial Hospital, Shandong University, Jinan, Shandong, China; b Department of Critical Care Medicine, The First Affiliated Hospital of Shandong First Medical University & Shandong Provincial Qianfoshan Hospital, Shandong medicine and Health Key Laboratory of Emergency Medicine, Shandong Institute of Anesthesia and Respiratory Critical Medicine, Jinan, Shandong, China; c Department of Nephrology, The Second Hospital of Shandong University, Shandong University, Jinan, Shandong, China.

**Keywords:** all-cause mortality, cardiovascular disease, chronic kidney disease, cohort study, riboflavin

## Abstract

Current studies have not clarified the relationship between riboflavin intake and all-cause mortality in patients with chronic kidney disease (CKD). The aim of this study is to investigate whether there is an association between riboflavin intake and the risk of all-cause mortality in patients with CKD. This was a retrospective cohort study with data extracted from the National Health and Nutrition Examination Survey (NHANES). The study was conducted using Cox regression analysis to calculate hazard ratio (HR) and 95% confidence interval (CI) to assess the association between riboflavin intake and risk of all-cause mortality. Subgroup analyses were performed regarding gender, CKD stage, hypertension, hyperlipidemia and cardiovascular disease (CVD). A total of 3750 patients were ultimately included in the analyses. After excluding potential confounders, lower intake of riboflavin was associated with the higher risk of all-cause mortality (Q1: HR = 1.33, 95% CI: 1.05–1.69). The similar association was also found in patients at mild/moderate stage (HR = 1.32, 95% CI: 1.05–1.66), in female (HR = 1.35, 95% CI: 1.01–1.81), with hypertension (HR = 1.37, 95% CI: 1.07–1.75), CVD (HR = 1.48, 95% CI: 1.08–2.03), and dyslipidemia (HR = 1.29, 95% CI: 1.01–1.66). This study found the association between low riboflavin intake and high risk of all-cause mortality, indicating a potential beneficial role of riboflavin in CKD patients.

## 1. Introduction

Chronic kidney disease (CKD) is a progressive disease characterized by structural and functional abnormalities of the kidneys for at least 3 months due to various causes.^[1]^ The global burden of CKD is substantial and growing: about 10% of adults worldwide are affected by some forms of CKD, resulting in 1.2 million deaths each year.^[1]^ It is estimated that CKD would be the fifth leading cause of death globally by 2040, which is 1 of the largest projected increases of any main causes of death.^[1]^ Therefore, identifying factors that affect the prognosis of CKD is of great significance for implementing reasonable interventions to reduce the burden of CKD.

Medical nutrition therapy is a critical aspect in medical intervention for CKD, as it alleviates disease symptoms, slows disease progression, and avoids the risk of malnutrition.^[2]^ Increasing evidence shows that the B-vitamin family is associated with CKD.^[3,4]^ Among them, riboflavin (vitamin B2) has received much attention as an essential vitamin and an essential enzyme cofactor.^[5]^ Riboflavin is an antioxidant nutrient that may have an independent antioxidant effect or as a component of the glutathione redox cycle.^[6]^ Oxidative stress can accelerate the progression of kidney diseases, and increased oxidative stress is associated with the several complications in patients at advanced stage of CKD.^[7]^ In addition, the deficiency of riboflavin can lead to an increase in homocysteine levels, thereby exacerbating the renal dysfunction.^[5,8]^ A study has reported that riboflavin intake lower than the recommended level was related to an elevated risk of CKD stage 3B or above.^[9]^ In the general populations, riboflavin intake was reported to show an inverse relationship with the risk of all-cause mortality.^[10]^ However, in CKD patients, the relationship between riboflavin intake and all-cause mortality has not been reported.

The aim of this study was to investigate whether riboflavin intake is associated with all-cause mortality in patients with CKD via a cohort study that provides support for the development of medical nutritional therapies for CKD patients.

## 2. Methods

### 2.1. Study design and data source

This study was a retrospective cohort study, written according to the STROBE statement guidelines, with data from the National Health and Nutrition Examination Survey (NHANES). NHANES is a survey combining interviews and physical examinations to assess the health and nutritional condition of American. Each year, the survey examined a representative sample of about 5000 persons. NHANES is a publicly available database and the information of participants are de-identified; therefore, this study didn’t require the informed consent and the ethical approval.

### 2.2. Study population

Participants were included: age ≥ 18 years old; diagnosed as CKD; and with complete data on dietary riboflavin intake in the NHANES 2007 to 2018. Participants were excluded: missing data on the survival; missing data on the baseline information; and with extreme total energy intakes (women: <500 or >5000 kcal/d, men: <500 or >8000 kcal/d).^[11]^

CKD was defined as urinary albumin to creatinine ratio > 30 mg/g and/or estimated glomerular filtration rate (eGFR) < 60 mL/min/1.73 m^2[12]^ and/or having medical history of CKD. CKD Epidemiology Collaboration creatinine equation was used for the calculation of eGFR. CKD stage was divided into mild/moderate (G1–G3) and severe/end-stage (G4–G5).^[13]^

Data on riboflavin intake were obtained using the 24-hour dietary recall interview, which was performed twice and requested participants to recall all the food and beverages consumed in the past 24 hours (midnight to midnight). In this study, we used the average value of the 24-hour dietary recalls, and riboflavin was divided into 3 group according to tertiles: Q1 (riboflavin < 1.518mg/d), Q2 (1.518 mg/d≤ riboflavin < 2.171 mg/d), Q3 (riboflavin ≥ 2.171 mg/d).

### 2.3. Data extraction

The data extracted in this study were based on demographic data, physical examination, comorbidities, history of medicine use, dietary intake, laboratory values, and outcome (all-cause mortality).

Demographic data included age, gender, race, educational level, marital status, poverty-to-income ratio (PIR), drinking status, smoking status, and physical activity. Physical examination included body mass index (BMI). Comorbidities included diabetes, dyslipidemia, hypertension, cancer, and cardiovascular disease (CVD). History of medicine use included angiotensin converting enzyme inhibitors (ACEI) and diuretic. Dietary intake included total energy intake, protein, calcium, sodium potassium ratio, vitamin D (D2 + D3), total fat, folate, and vitamin B12. Laboratory values included white blood cell (WBC) count, hemoglobin (HGB), platelet count (PLT), alkaline phosphatase (ALP), gamma-glutamyl transaminase (GGT), uric acid, aspartate aminotransferase (AST), and alanine aminotransferase (ALT).

BMI was determined based on the following calculation formula: body weight (kg)/height (m)^2^. Diabetes was determined as glycated hemoglobin ≥ 6.5%, or fasting blood glucose ≥ 7.0 mmol/L, or self-reported diabetes, or self-reported hypoglycemic treatment. Hypertension was determined as diastolic blood pressure ≥ 80 mm Hg, or systolic blood pressure ≥ 130 mm Hg, or self-reported hypertension, or self-reported use of antihypertensive medications. Dyslipidemia was defined as total cholesterol ≥ 200 mg/dL, or triglyceride ≥ 150 mg/dL, or low-density lipoprotein cholesterol ≥ 130 mg/dL, or high-density lipoprotein cholesterol ≤ 40 mg/dL, or self-reported hypercholesterolemia, or receiving cholesterol lowering treatment. All-cause mortality was defined as death from any cause.^[14]^

### 2.4. Statistical analysis

The appropriate sample weights provided by NHANES were used to weight the data to account for complex sampling design. The continuous data were shown as mean (standard error) (SE), and *t*-test was used for the comparison of differences between 2 groups. The categorical data were shown as number and percentage [(n) %], and χ^2^ test was used for comparison of differences between 2 groups. The difference analysis was used to select covariates. Cox regression analysis was used to assess the relationship between riboflavin intake and all-cause mortality. Results were reported as hazard ratio (HR) and 95% confidence interval (CI). Subgroup analysis was performed according to gender, CKD stage, hypertension, hyperlipidemia, and CVD. The statistical analysis was conducted using SAS 9.4 (SAS Institute Inc., Cary, NC, USA), and *P* < .05 was regarded as significant difference.

## 3. Results

### 3.1. Selection and characteristics of included patients

**Figure 1.**
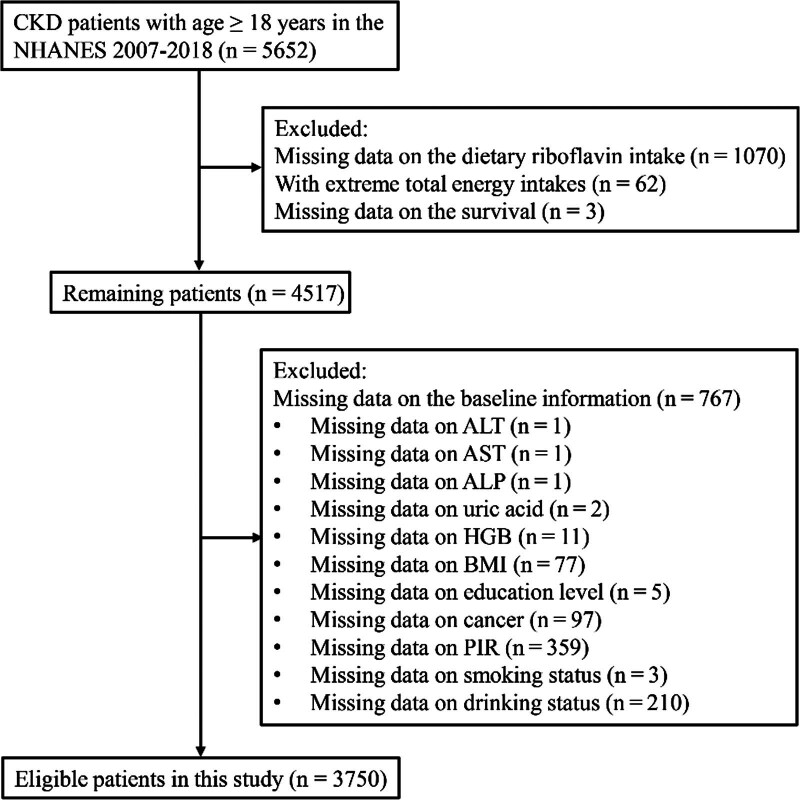
The flowchart of the selection of patients.

A total of 5652 CKD patients aged ≥ 18 years were extracted. Further, 1070 patients missing data on the dietary riboflavin intake, 62 patients with extreme total energy intakes, and 3 patients missing data on the survival were extracted. In the remaining 4517 patients, 767 patients missing data on the baseline information were excluded. Finally, 3750 patients were included for analysis (Fig. 1). Table 1 summarizes the characteristics of included patients. There were 2784 patients in the survival group and 966 patients in the all-cause mortality group. The mean age of total patients was 60.54 years, and 1781 (43.89%) patients were male. There was significant difference in age, gender, race, education level, marital status, drinking status, physical activity, diabetes, dyslipidemia, hypertension, cancer, CVD, ACEI, diuretic, total energy intake, protein, calcium, HGB, ALP, uric acid, and CKD stage between the survival group and all-cause mortality group.

**Table 1:** Baseline information of CKD patients in the survival group and all-cause mortality group.

Variables	Total (n = 3750)	Survival group (n = 2784)	All-cause mortality group (n = 966)	Statistics	*P*
Age, yr, Mean (SE)	60.54 (0.39)	57.67 (0.46)	71.51 (0.57)	*t* = −19.31	<.001
Gender, n (%)				χ^2^ = 6.062	.014
Female	1969 (56.11)	1537 (57.43)	432 (51.08)		
Male	1781 (43.89)	1247 (42.57)	534 (48.92)		
Race, n (%)				χ^2^ = 42.433	<.001
Mexican American	442 (6.65)	376 (7.59)	66 (3.03)		
Non-Hispanic Black	813 (10.88)	627 (11.20)	186 (9.63)		
Non-Hispanic White	1939 (72.50)	1307 (70.20)	632 (81.30)		
Other Hispanic	290 (3.70)	244 (4.14)	46 (2.02)		
Other races	266 (6.28)	230 (6.86)	36 (4.03)		
Education level, n (%)				χ^2^ = 33.490	<.001
9th to 11th grades	572 (11.98)	391 (10.75)	181 (16.68)		
High school grade/GED or equivalent	954 (25.66)	695 (25.33)	259 (26.92)		
Less than 9th grade	448 (7.33)	300 (6.11)	148 (12.02)		
More than high school	1776 (55.03)	1398 (57.81)	378 (44.38)		
Marital status, n (%)				χ^2^ = 6.212	.013
Married	1922 (54.41)	1462 (55.82)	460 (48.98)		
Unmarried	1828 (45.59)	1322 (44.18)	506 (51.02)		
PIR, n (%)				χ^2^ = 0.345	.557
<1	764 (15.22)	571 (15.00)	193 (16.07)		
≥1	2986 (84.78)	2213 (85.00)	773 (83.93)		
Drinking status, n (%)				χ^2^ = 7.196	.007
No	1270 (29.42)	922 (27.95)	348 (35.05)		
Yes	2480 (70.58)	1862 (72.05)	618 (64.95)		
Smoking status, n (%)				χ^2^ = 0.037	.847
No	3026 (81.05)	2246 (81.16)	780 (80.64)		
Yes	724 (18.95)	538 (18.84)	186 (19.36)		
Physical activity, n (%)				χ^2^ = 118.407	<.001
≤ 450MET*min/wk	416 (12.17)	328 (12.86)	88 (9.52)		
> 450MET*min/wk	1833 (54.12)	1510 (59.22)	323 (34.59)		
Unknown	1501 (33.70)	946 (27.91)	555 (55.89)		
BMI, kg/m^2^, Mean (SE)	30.49 (0.21)	30.52 (0.25)	30.34 (0.39)	*t* = 0.40	.688
Diabetes, n (%)				χ^2^ = 37.724	<.001
No	2279 (67.18)	1752 (70.29)	527 (55.24)		
Yes	1471 (32.82)	1032 (29.71)	439 (44.76)		
Dyslipidemia, n (%)				χ^2^ = 35.414	<.001
No	578 (16.27)	471 (18.30)	107 (8.50)		
Yes	3172 (83.73)	2313 (81.70)	859 (91.50)		
Hypertension, n (%)				χ^2^ = 94.388	<.001
No	572 (20.03)	516 (23.86)	56 (5.36)		
Yes	3178 (79.97)	2268 (76.14)	910 (94.64)		
Cancer, n (%)				χ^2^ = 43.528	<.001
No	3061 (80.98)	2354 (84.02)	707 (69.35)		
Yes	689 (19.02)	430 (15.98)	259 (30.65)		
CVD, n (%)				χ^2^ = 86.073	<.001
No	2357 (68.59)	1942 (74.20)	415 (47.05)		
Yes	1393 (31.41)	842 (25.80)	551 (52.95)		
ACEI, n (%)				χ^2^ = 8.521	.004
No	2683 (74.17)	2040 (75.85)	643 (67.75)		
Yes	1067 (25.83)	744 (24.15)	323 (32.25)		
Diuretic, n (%)				χ^2^ = 60.683	<.001
No	2548 (72.58)	2016 (76.73)	532 (56.65)		
Yes	1202 (27.42)	768 (23.27)	434 (43.35)		
Total energy intake, kcal, Mean (SE)	1907.30 (16.69)	1945.39 (20.13)	1761.31 (27.70)	*t* = 5.29	<.001
Protein, g, Mean (SE)	75.27 (0.74)	76.70 (0.87)	69.80 (1.28)	*t* = 4.42	<.001
Calcium, mg, Mean (SE)	875.80 (10.62)	894.26 (12.40)	805.05 (18.24)	*t* = 4.06	<.001
Sodium potassium ratio, %, Mean (SE)	1.32 (0.01)	1.32 (0.01)	1.29 (0.02)	*t* = 1.18	.243
Vitamin D, mcg, Mean (SE)	4.60 (0.13)	4.61 (0.15)	4.54 (0.19)	*t* = 0.30	.764
Total fat, g, Mean (SE)	165.55 (3.88)	166.15 (4.60)	163.26 (5.28)	*t* = 0.43	.670
Folate, DFE, mcg, Mean (SE)	484.11 (7.79)	489.68 (9.12)	462.75 (11.52)	*t* = 1.90	.060
Vitamin B12, mcg, Mean (SE)	4.79 (0.10)	4.82 (0.12)	4.69 (0.15)	*t* = 0.71	.481
WBC count, 1000 cells/uL, Mean (SE)	7.68 (0.10)	7.62 (0.12)	7.92 (0.15)	*t* = −1.59	.114
HGB, g/dL, Mean (SE)	13.84 (0.05)	13.95 (0.05)	13.44 (0.10)	*t* = 4.94	<.001
PLT, 1000 cells/uL, Mean (SE)	238.50 (1.99)	240.75 (2.06)	229.89 (5.83)	*t* = 1.75	.084
ALP, U/L, Mean (SE)	73.17 (0.80)	72.34 (0.89)	76.39 (1.55)	*t* = −2.37	.020
GGT, U/L, Mean (SE)	33.62 (1.77)	30.95 (1.24)	43.85 (6.92)	*t* = −1.84	.069
Uric acid, mg/dL, Mean (SE)	6.01 (0.04)	5.91 (0.05)	6.38 (0.08)	*t* = −5.02	<.001
AST, U/L, Mean (SE)	26.96 (0.58)	26.27 (0.55)	29.57 (1.80)	*t* = −1.76	.081
ALT, U/L, Mean (SE)	24.90 (0.77)	24.84 (0.61)	25.12 (2.59)	*t* = −0.11	.915
CKD stage, n (%)				χ^2^ = 75.851	<.001
Mild/moderate	3562 (96.67)	2689 (97.98)	873 (91.62)		
Severe/end-stage	188 (3.33)	95 (2.02)	93 (8.38)		
Riboflavin, n (%)				χ^2^ = 1.635	.441
Q1	1409 (33.26)	1061 (32.66)	348 (35.59)		
Q2	1217 (33.38)	909 (33.90)	308 (31.35)		
Q3	1124 (33.36)	814 (33.44)	310 (33.06)		

Abbreviations: ACEI = angiotensin converting enzyme inhibitors, ALP = alkaline phosphatase, ALT = alanine aminotransferase, AST = aspartate aminotransferase, BMI = body mass index, CKD = Chronic kidney disease, CVD = cardiovascular disease, DFE = dietary folate equivalent, GED = General Educational Development, GGT = gamma-glutamyl transaminase, HGB = hemoglobin, MET = metabolic equivalent of task, PIR = poverty-to-income ratio, PLT = platelet count, WBC = white blood cell.

### 3.2. Association between riboflavin and all-cause mortality of CKD patients

In the crude model, no significant difference was found between riboflavin intake and the risk of all-cause mortality in CKD patients (Q1: HR = 1.17, 95% CI: 0.92–1.48; Q3: HR = 0.99, 95% CI: 0.79–1.25). After adjusting age, gender, race, education level, marital status, drinking status, physical activity, diabetes, dyslipidemia, hypertension, cancer, CVD, ACEI, diuretic, total energy intake, protein, calcium, HGB, ALP, uric acid, and CKD stage, we found that lower intake of riboflavin had a significant relationship with the higher risk of all-cause mortality (Q1: HR = 1.33, 95% CI: 1.05–1.69) (Table 2).

**Table 2:** Association between riboflavin intake and the risk of all-cause mortality. Riboflavin was divided into 3 group according to tertiles: Q1 (riboflavin < 1.518 mg/d), Q2 (1.518 mg/d ≤ riboflavin < 2.171 mg/d), Q3 (riboflavin ≥ 2.171 mg/d).

Riboflavin intake	Model 1		Model 2	
	HR (95% CI)	P	HR (95% CI)	*P*
Q1	1.17 (0.92–1.48)	0.193	1.33 (1.05–1.69)	.019
Q2	Ref		Ref	
Q3	0.99 (0.79–1.25)	0.960	1.15 (0.86–1.55)	.336

Abbreviations: CI = confidence interval, HR = hazard ratio.

Model 1: crude model; model 2: adjusting age, gender, race, education level, marital status, drinking status, physical activity, diabetes, dyslipidemia, hypertension, cancer, CVD, ACEI, diuretic, total energy intake, protein, calcium, HGB, ALP, uric acid, CKD stage.

### 3.3. Subgroup analysis

**Figure 2.**
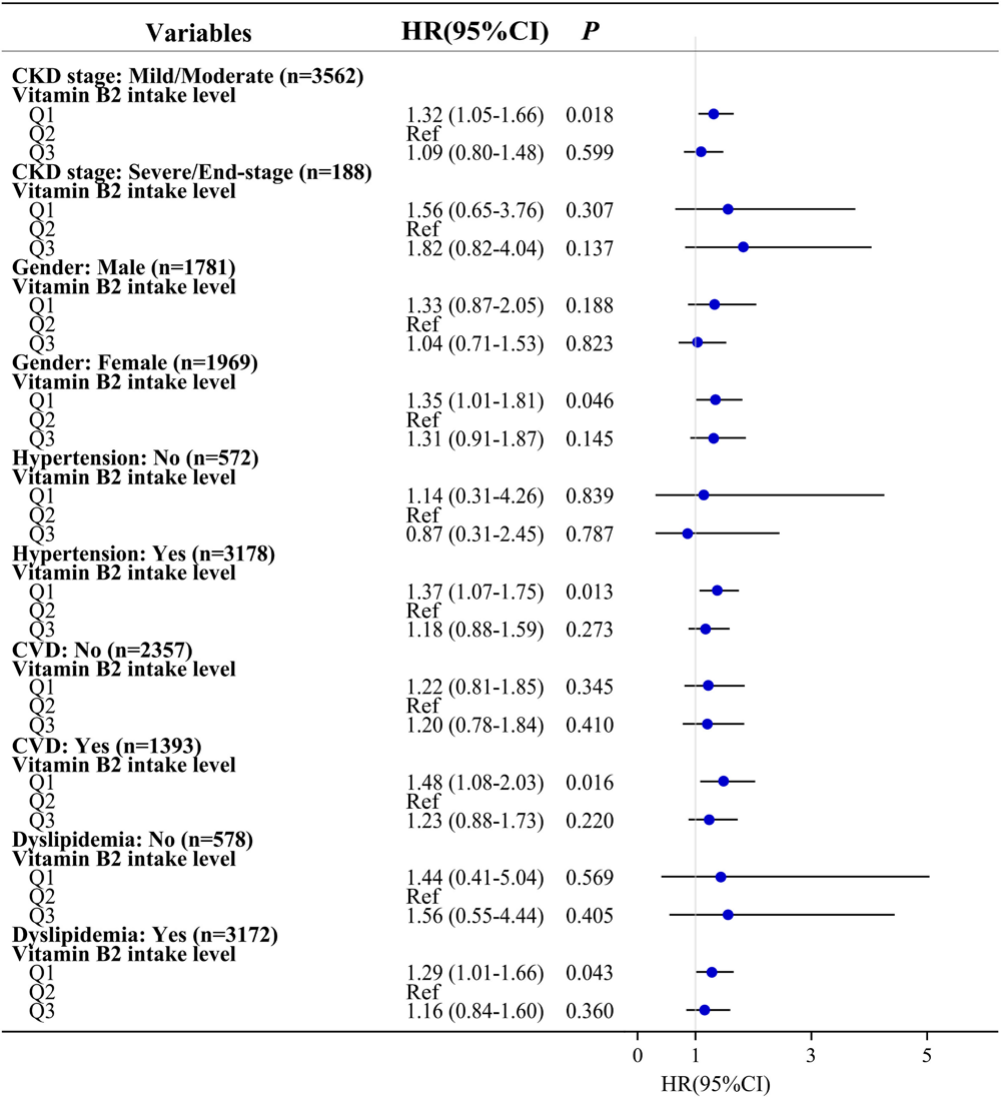
Subgroup analysis for the association between riboflavin intake and all-cause mortality of CKD patients. Riboflavin was divided into 3 group according to tertiles: Q1 (riboflavin < 1.518mg/d), Q2 (1.518 mg/d ≤ riboflavin < 2.171 mg/d), Q3 (riboflavin ≥ 2.171mg/d). Abbreviations: CKD, chronic kidney disease; CVD, cardiovascular disease.

For subgroup analysis, age, gender (or not), race, education level, marital status, drinking status, physical activity, diabetes, dyslipidemia (or not), hypertension (or not), cancer, CVD (or not), ACEI, diuretic, total energy intake, protein, calcium, HGB, ALP, uric acid, and CKD stage (or not) were adjusted. In the CKD stage subgroup, lower intake of riboflavin was associated with high risk of all-cause mortality in patients at mild/moderate stage of CKD (HR = 1.32, 95% CI: 1.05–1.66). In the gender subgroup, we found that lower riboflavin intake was related to the higher risk of all-cause mortality in females (HR = 1.35, 95% CI: 1.01–1.81). In the hypertension subgroup, the significant association between riboflavin intake and all-cause mortality was found in patients with hypertension (HR = 1.37, 95% CI: 1.07–1.75). In the CVD subgroup, lower intake of riboflavin was correlated with higher risk of all-cause mortality in patients with CVD (HR = 1.48, 95% CI: 1.08–2.03). In the dyslipidemia subgroup, we found the significant association between lower riboflavin intake and higher risk of all-cause mortality in patients with dyslipidemia (HR = 1.29, 95% CI: 1.01–1.66) (Fig. 2).

## 4. Discussion

In this retrospective cohort study, we explored whether riboflavin intake had a relationship with the prognosis of CKD patients using NHANES database which included nationally representative data in the USA. We found a significant association between low intake of riboflavin and high risk of all-cause mortality. The similar association was found in patients at mild/moderate stage of CKD, in females, with hypertension, with CVD, and with dyslipidemia. Our results indicated that increasing the intake of riboflavin might improve the prognosis of CKD patients.

CKD has been reported to bring substantial burden and leads to 1.2 million deaths annually.^[1]^ In this study, we found that low intake of riboflavin showed an association with the high risk of all-cause mortality in CKD patients. There were several explanations for this association. One potential mechanism was oxidative stress.^[7,15]^ Riboflavin was an antioxidant nutrient that may have an antioxidant effect independently or as a component of the glutathione redox cycle.^[15]^ Due to antioxidant depletions and increased reactive oxygen production, oxidative stress was reported in kidney diseases.^[7]^ The kidney is a metabolically active organ and has mitochondria that are rich in oxidative reactions, making it susceptible to damage from oxidative stress.^[16]^ Low intake of riboflavin might increase the oxidative stress.^[15]^ Several studies have shown that oxidative stress can accelerate the progression of kidney diseases.^[17,18]^ Also, in CKD patients, increased oxidative stress was associated with some complications such as hypertension, atherosclerosis, inflammation, and anemia, which elevated the risk of death.^[7,19]^ Another potential explanation was the increase of homocysteine level.^[5]^ B-vitamin family has been reported as an established predictor of total homocysteine plasma level in the CKD patients.^[20]^ Plasma total homocysteine levels are elevated in most patients with renal injury.^[21]^ Evidence has shown that low riboflavin (vitamin B2) level was associated with the high plasma level of fasting total homocysteine in patients with end-stage renal disease.^[5]^ A study has reported that high total homocysteine level may worsen the kidney function, thereby elevating the risk of death.^[22]^

Gender has been reported to influence the progression of CKD.^[23]^ The prevalence of CKD was higher in females, whereas there was fewer females on renal replacement therapy than males.^[23]^ Males may develop end-stage renal disease faster.^[23]^ In this study, we found that low intake of riboflavin had a relationship with the increased risk of all-cause mortality in females, indicating the importance of increasing riboflavin intake for females to improve the prognosis of CKD patients. Further, we found the association between low intake of riboflavin and high risk of all-cause mortality in CKD patients at mild/moderate stage, while no association was found in patients at severe/end-stage. This may be explained by that the risk of death increases exponentially as kidney function worsens in patients at severe/end-stage,^[24]^ which may weaken the relationship between riboflavin and death. In addition, the sample size of patients at severe/end-stage is relatively small (n = 188), which may not have enough statistical power to support us to obtain significant results.

CKD was strongly associated with the high risk of CVD.^[25]^ The association is multifactorial, but chronic renal failure is usually associated with dyslipidemia, which may be 1 of the possible causes.^[25]^ In addition, Pugh et al reported that controlling hypertension was important to slow the disease progression and reduce CVD risk in CKD patients.^[26]^ In this study, we found that low intake of riboflavin was significantly associated with the high risk of all-cause mortality in CKD patients with hypertension, dyslipidemia, or CVD. Several studies have shown that riboflavin supplementation could lower the blood pressure levels in hypertensive patients.^[27,28]^ A study has reported the intake of riboflavin was inversely associated with the odds of dyslipidemia^[29]^ Liu et al reported that low intake of riboflavin increased the risk of hypertension,^[30]^ thereby increasing the risk of death.

This study used the representative samples from NHANES database to investigate the relationship between riboflavin and all-cause mortality in CKD patients, and found that low riboflavin intake was associated with the high risk of all-cause mortality. Our findings emphasized the role of riboflavin in the prognosis of CKD. However, there are some limitations in this study. First, it is difficult to obtain the data in the follow-up period due to the limitation in the investigation method of the database, which may cause some bias. Secondly, the ethnicity, economic level, lifestyle and other factors of people with CKD vary in different countries. Therefore, our findings need to be further validated in patients with CKD in other countries.

## 5. Conclusion

In this study, we found that low riboflavin intake was associated with a high risk of all-cause mortality in patients with CKD. Our study supports the potential beneficial effects of riboflavin in patients with CKD. The results of this study provide a foundation for further understanding of the impact of riboflavin on the prognosis of patients with CKD.

## Author contributions

**Conceptualization:** Xiaoxu Ren, Rong Wang, Zhiming Jiang.

**Data curation:** Xiaoxu Ren, Hairong Chen.

**Formal analysis:** Xiaoxu Ren.

**Investigation:** Rong Wang, Fen Liu.

**Methodology:** Fen Liu, Quanzhen Wang.

**Resources:** Yunfeng Hou, Lifeng Yu.

**Software:** Quanzhen Wang, Yunfeng Hou.

**Supervision:** Zhiming Jiang.

**Validation:** Hairong Chen, Lifeng Yu.

**Visualization:** Hairong Chen, Lifeng Yu.

**Writing – original draft:** Xiaoxu Ren.

**Writing – review & editing:** Xiangchun Liu, Zhiming Jiang.
